# Supplementary material for: Autophagy inhibition rescues structural and functional defects caused by the loss of mitochondrial chaperone Hsc70-5 in Drosophila
Source: Autophagy. 2021 Jan 25;17(10):3160–74. doi: 10.1080/15548627.2020.1871211 (PMC8526020; doi:10.1080/15548627.2020.1871211)
Supplement: Supplemental Material [file KAUP_A_1871211_SM4946.zip › supplement/figure s1.pdf]

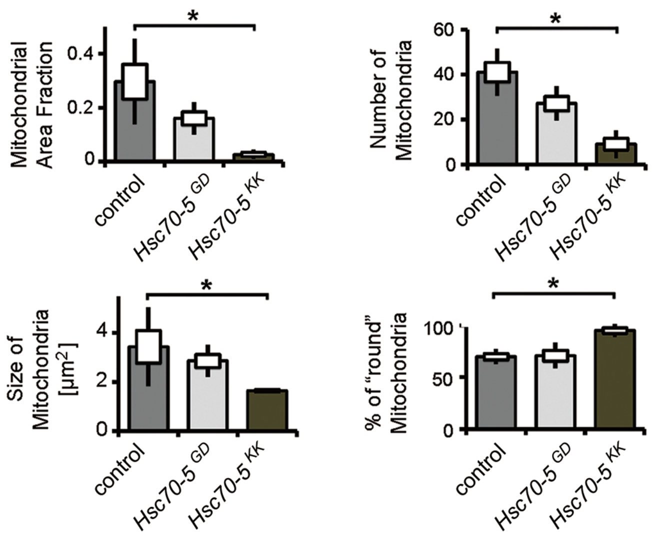

Mitochondrial Area Fraction
0.4
0.2
0
*
control
Hsc70-5 GD
Hsc70-5 KK
Number of Mitochondria
60
40
20
0
*
control
Hsc70-5 GD
Hsc70-5 KK
Size of Mitochondria [μm2]
4
2
0
*
control
Hsc70-5 GD
Hsc70-5 KK
% of "round" Mitochondria
100
50
0
*
control
Hsc70-5 GD
Hsc70-5 KK
