# Supplementary material for: Autophagy inhibition rescues structural and functional defects caused by the loss of mitochondrial chaperone Hsc70-5 in Drosophila
Source: Autophagy. 2021 Jan 25;17(10):3160–74. doi: 10.1080/15548627.2020.1871211 (PMC8526020; doi:10.1080/15548627.2020.1871211)
Supplement: Supplemental Material [file KAUP_A_1871211_SM4946.zip › supplement/Supplementary information_R3.docx]

**Supplementary information**

**Figure legends for supplementary figures**

**Figure S1**. Quantification of mitochondrial parameters in *Hsc70-5* knockdown larvae. Quantification of mitochondrial area fraction, number, size and shape in control, *Hsc70-5^GD^* and *Hsc70-5^KK^* larvae. Standard error of mean and standard deviation are shown as a box and a black line. * p<0.05.

**Figure S2**. Quantification of synaptic terminals in *Hsc70-5* knockdown larvae. (**A**) Confocal images of NMJ labeled with hrp-Cy3. Scale bar: 5 μm, Enlargement: 2 μm. (**B**) Quantification of muscle length, NMJ size, number, and bouton size of synaptic boutons in control, *Hsc70-5^GD^* and *Hsc70-5^KK^* larvae. Standard error of mean and standard deviation are shown as a box and a black line.

**Figure S3**. Quantification of futsch staining in nerve terminals. (**A**) futsch loops per terminal end and (**B**) % NMJ area innervated by futsch in indicated genotypes. Standard error of mean and standard deviation are shown as a box and a black line. * p<0.05.
