# Supplementary material for: Autophagy inhibition rescues structural and functional defects caused by the loss of mitochondrial chaperone Hsc70-5 in Drosophila
Source: Autophagy. 2021 Jan 25;17(10):3160–74. doi: 10.1080/15548627.2020.1871211 (PMC8526020; doi:10.1080/15548627.2020.1871211)
Supplement: Supplemental Material [file KAUP_A_1871211_SM4946.zip › supplement/Table S1 R3.docx]

**Table S1**. Categorization of pathological stages in larval and adult stages.

| **Stages** | **Model** | **Genotype/time point of analysis** | **Conditions** | **Locomotion phenotype** |
| --- | --- | --- | --- | --- |
| Larva | (A) pre-symptomatic | elav>*Hsc70-5^GD13957^*/L3 | 25°C | Crawling velocity and righting reflex unchanged |
|  | (B) symptomatic | elav>*Hsc70-5^KK100233^*/L3 | 25°C | Crawling velocity lower and righting reflex slower |
|  |  | elav>*Hsc70-5^KK100233^,tub-Gal80^ts^*/L3 | 1 d at 18°C → 25°C | Righting reflex slower |
| Adult | (A) symptomatic | elav>*Hsc70-5^KK100233^,tub-Gal80^ts^*/5-d-old | 5 d at 18°C → 25°C | Climbing efficiency reduced |
|  | (B) late-symptomatic | elav>*Hsc70-5^KK100233^,tub-Gal80^ts^*/10-d-old | 5 d at 18°C → 25°C | Exacerbated climbing defects |
|  |  |  |  |  |
|  |  |  |  |  |

Presymptomatic larvae: L3 elav>*Hsc70-5^GD13957^* larvae had no detectable locomotion defects compared to control and were categorized as presymptomatic. Symptomatic larvae: L3 elav>*Hsc70-5^KK100233^* larvae displayed lower crawling velocity and slower righting reflex compared to control and were classified as symptomatic. Similarly, elav>*Hsc70-5^KK100233^,tub-Gal80^ts^* L3 larvae were also classified as symptomatic due to a slower righting reflex compared to age-matched control larvae. Symptomatic adult: elav>*Hsc70-5^KK100233^,tub-Gal80^ts^* flies at 4 d were referred to as symptomatic based on their impaired climbing ability compared to control flies. Late-symptomatic larvae: At 10 d, elav>*Hsc70-5^KK100233^,tub-Gal80^ts^* flies displayed a more severe climbing defect compared to control and perform worse than 4-d-old elav>*Hsc70-5^KK100233^,tub-Gal80^ts^*. They were categorized as late-symptomatic.
