# Supplementary material for: Autophagy inhibition rescues structural and functional defects caused by the loss of mitochondrial chaperone Hsc70-5 in Drosophila
Source: Autophagy. 2021 Jan 25;17(10):3160–74. doi: 10.1080/15548627.2020.1871211 (PMC8526020; doi:10.1080/15548627.2020.1871211)
Supplement: Supplemental Material [file KAUP_A_1871211_SM4946.zip › supplement/Table S2.docx]

**Table S2**. Fly strains.

| **Genes** | **CG number** | **Strain** |
| --- | --- | --- |
| *Atg1* | CG10967 | BL-26731 |
| *Atg101* | CG7053 | BL-34360 |
| *Vps15* | CG9746 | BL-35209 |
| *Buffy* | CG8238 | BL-32060 |
| *Atg5* | CG1643 | BL-27551 |
| *Atg7* | CG5489 | BL-27707 |
| *Atg8* | CG32672 | BL-34340 |
| *Atg12* | CG10861 | BL-27552 |
| *Snap25* | CG40452 | BL-27306 |
| *Sytβ* | CG42333 | BL-27293 |
| *Syx7* | CG5081 | BL-29546 |
| *brp* | CG42344 | UAS-Brp-RNAi from Stephan Sigrist (Wagh et al., 2006) |
| *VGlut* | CG9887 | BL-27538 |
| *Rph* | CG11556 | BL-25950 |
| *Rop* | CG15811 | BL-28929 |
| *Sec15* | CG7034 | BL-27499 |
| *EndoA* | CG14296 | BL-27679 |
| *Eps-15* | CG16932 | BL-29578 |
| *Dj-1β* | CG6646 | BL-31261 |
| *Lrrk* | CG5483 | BL-32457 |
| *DCTN2-p50* | CG8269 | BL-28596 |
| *DCTN1-p150* | CG9206 | BL-27721 |
| *tko* | CG7925 | BL-38251 |
|  |  |  |
| **Others** | | **Strain** |
| *White RNAi* | | VDRC-GD30033 |
| *Hsc70-5* RNAi | | VDRC-GD47745 |
| *Hsc70-5* RNAi | | VDRC-KK106236 |
| Gal80^ts^ | | BL-7019 |
| UAS-*Dicer* | | BL-24646 |
| UAS-*Atg1* | | Thomas Neufeld (University of Minnesota) |
| hs-Flp;UAS-Dcr2;R4-mCherry-*Atg8a*,Act>CD2>Gal4,UAS-GFPnls | | mosaic stock from Gábor Juhász (Eotvos Lorand University) |
| *Gmr*-Gal4 | | Aaron Voigt (University Clinic Aachen) |
| *lmd-RNAi* | | Aaron Voigt (University Clinic Aachen) |
| UAS-*Pink1* | | Ming Guo (University of California Los Angeles) |
