# Supplementary material for: Autophagy inhibition rescues structural and functional defects caused by the loss of mitochondrial chaperone Hsc70-5 in Drosophila
Source: Autophagy. 2021 Jan 25;17(10):3160–74. doi: 10.1080/15548627.2020.1871211 (PMC8526020; doi:10.1080/15548627.2020.1871211)
Supplement: Supplemental Material [file KAUP_A_1871211_SM4946.zip › supplement/Zhu, Hannan et al., 2020 Manuscript_R2_CP.docx]

**Autophagy inhibition rescues structural and functional defects caused by the loss of mitochondrial chaperone *Hsc70-5* in *Drosophila***

**Running Title:**

**Autophagy inhibition alleviates mitochondria-associated loss of Hsc70-5 function in *Drosophila***

Jun-yi Zhu^1,2,*^, Shabab B. Hannan^1,3,*^, Nina M. Dräger^3^, Natalia Vereshchagina^1^, Ann-Christin Krahl^1^, Yulong Fu^2^, Christopher J.H. Elliott^4^, Zhe Han^2^, Thomas R. Jahn^3,6^ & Tobias M. Rasse^1,3,5^

^1^Hertie Institute for Clinical Brain Research, University of Tübingen, Otfried-Müller-Str. 27, 72076 Tübingen, Germany

^2^Center for Genetic Medicine Research, Children's National Medical Center, Washington, DC 20010, USA

^3^Schaller Research Group at the University of Heidelberg and DKFZ, Proteostasis in Neurodegenerative Disease (B180), German Cancer Research Center, Im Neuenheimer Feld, 69120 Heidelberg, Germany

^4^Department of Biology, University of York, York, YO1 5DD, UK

^5^Max Planck Institute for Heart and Lung Research, Ludwigstr. 43, 61231 Bad Nauheim, Germany

^6^Present address: AbbVie Deutschland GmbH, Knollstr., 67061 Ludwigshafen, Germany

^*^These authors contributed equally to this work.

Correspondence: tobias.rasse@mpi-bn.mpg.de

**Keywords:** *Atg1, Hsc70-5,* microtubule*,* mitochondria, mitophagy, rapamycin, synapse

**Abstract**

We investigated in larval and adult *Drosophila* models whether loss of the mitochondrial chaperone *Hsc70-5* is sufficient to cause pathological alterations commonly observed in Parkinson disease. At affected larval neuromuscular junctions, no effects on terminal size, bouton size or number, synapse size, or number were observed, suggesting that we studied an early stage of pathogenesis. At this stage, we noted a loss of synaptic vesicle proteins and active zone components, delayed synapse maturation, reduced evoked and spontaneous excitatory junctional potentials, increased synaptic fatigue, and cytoskeleton rearrangements. The adult model displayed ATP depletion, altered body posture, and susceptibility to heat-induced paralysis. Adult phenotypes could be suppressed by knockdown of *dj-1β*, *Lrrk*, *DCTN2-p50*, *DCTN1-p150*, *Atg1*, *Atg101*, *Atg5*, *Atg7,* and *Atg12*. The knockdown of components of the macroautophagy/autophagy machinery or overexpression of human *HSPA9* broadly rescued larval and adult phenotypes, while disease-associated *HSPA9* variants did not. Overexpression of *Pink1* or promotion of autophagy exacerbated defects.

**Abbreviations:** AEL: after egg laying; AZ: active zone; brp: bruchpilot; Csp: cysteine string protein; dlg: discs large; eEJPs: evoked excitatory junctional potentials; GluR: glutamate receptor; H_2_O_2_: hydrogen peroxide; mEJP: miniature excitatory junctional potentials; MT: microtubule; NMJ: neuromuscular junction; PD: Parkinson disease; *Pink1*: PTEN-induced putative kinase 1; PSD: postsynaptic density; SSR: subsynaptic reticulum; SV: synaptic vesicle; VGlut: vesicular glutamate transporter.

**Introduction**

Parkinson disease (PD), the most prevalent movement disorder and the second most prevalent neurodegenerative disease, is characterized by resting tremor, stiffness, and slowness of movement. Analysis of genetic and environmental factors contributing to PD suggests that impairments in mitochondrial function, lysosomal degradation pathways, and synaptic transmission are of central importance to pathogenesis and progression of PD [1-4]. Due to their complex morphology and high-energy demands, neurons in the adult brain are particularly susceptible to dysregulation of mitochondrial quality control systems. These quality control systems operate at different levels protecting cells and tissues from dysfunctional mitochondria. While a significant proportion of PD cases are sporadic, mutations in at least 11 genes have been implicated in monogenic typical or atypical forms of parkinsonism [3], providing crucial insights into the cellular and molecular pathways involved in PD. In all cases, the detailed molecular mechanisms leading to disease development have not been fully elucidated. However, a number of PD-associated genes have been implicated in mitochondrial function, altered mitochondrial dynamics, or the accumulation of dysfunctional mitochondria, which are characteristic features of PD [6-11]. For example, the PD-associated genes *Pink1* (PTEN-induced kinase 1) and *park* are important regulators of mitochondrial quality control and mitophagy, a form of macroautophagy [6-8]. Mitochondrial dysfunctions have also been implicated in various other neurodegenerative diseases such as Alzheimer disease where mitochondria are key targets of both Aβ42 and tau toxicity [12].

We have previously addressed the importance of mitochondrial quality control and selective vulnerability of dopaminergic neurons by generating a model for loss of function of *Drosophila* *Hsc70-5* [13]. Enhanced mitophagy is identified as an early pathological feature caused by knockdown in a presymptomatic model of loss of *Hsc70-5* function [13] prior to the emergence of locomotion defects. Consistent with the upregulation of mitophagy upon loss of Hsc70-5 function in a *Drosophila*, human fibroblasts obtained from a carrier of the PD-associated HSPA9^A476T^ variant exhibit increased colocalization of mitochondria with autophagosomes.

In the current study, we investigated in detail the consequences of neuronal loss of Hsc70-5 function *in vivo* and examine epistatic interactions in this genetic background.

**Results**

***Impairment of larval locomotion upon loss of Hsc70-5 function.***

We have previously shown that strong pan-neuronal expression (*elav-Gal4, 29°C*) of the RNAi-construct *Hsc70-5^KK100233^* results in lethality at the late L3 larval stage [14]. In the current study, we sought to investigate cellular and functional perturbations that occur early in pathogenesis. We thus reduced silencing potency by raising larvae at 25°C, which delayed lethality to the pupal stage (elav>*Hsc70-5^KK100233^* and elav>*Hsc70-5^GD13957^*). elav>*Hsc70-5^KK100233^* larvae were sluggish compared to size-matched elav>*Hsc70-5^GD13957^* and control larvae. Their crawling velocity was reduced and the righting reflex delayed (**Fig. 1A**). elav>*Hsc70-5^GD13957^* larvae were indistinguishable from controls in their righting ability. Hence we referred to them as presymptomatic and elav>*Hsc70-5^KK100233^* larvae as symptomatic (**Table S1**). *Hsc70-5* is an important regulator of mitochondrial function [13,15-17]. Thus, we investigated putative mitochondrial impairments in presymptomatic and symptomatic larvae. Pan-neuronal *Hsc70-5* knockdown resulted in severe reductions in mitochondrial mass at neuromuscular junctions (NMJs) of symptomatic (elav>*Hsc70-5^KK100233^*, 25°C) but not presymptomatic (elav>*Hsc70-5^GD13957^*, 25°C) larvae compared to control (**Fig. 1B**). Symptomatic larvae showed a reduction in mitochondrial number and size, and consequently, a decrease in mitochondria area fraction at the NMJ (**Fig. S1**). Muscle length, NMJ area, and bouton numbers were unaltered in both presymptomatic and symptomatic larvae (**Fig. S2**). This suggested that elav>*Hsc70-5^KK100233^* larvae represented an early stage in disease progression characterized by the absence of gross neurodevelopmental or neurodegeneration phenotypes.

***Detection of early synaptic changes due to loss of Hsc70-5 function.***

To further investigate structural changes in response to loss of Hsc70-5 function, we stained for synaptic vesicles (SVs) and active zone (Az) markers. To this aim, we performed confocal imaging on fixed larval NMJs. Analysis and quantification revealed that SV proteins Csp (cysteine-string protein, **Fig. 1C**) and VGlut (vesicular glutamate transporter, **Fig. 1D**) were reduced at NMJs of symptomatic larvae compared to control larvae. A similar reduction was observed for the central organizing component of Azs, brp (bruchpilot, **Fig. 1E**). We observed no significant differences in presymptomatic larvae for SV proteins or Az components compared to control (**Fig. 1C-E**).

We proceeded to investigate synapse maturation by analyzing subsynaptic reticulum (SSR) and postsynaptic density (PSD) maturation. The SSR, a complex system of membrane tubules and lamellae, is formed by the invagination of postsynaptic membranes following presynaptic innervation. Thus, an increased percentage of terminal synaptic boutons devoid of SSR can be used as a marker for delayed synapse terminal maturation [18,19]. To examine SSR folding and morphogenesis, we labeled NMJs with antibodies against dlg and the neuronal marker hrp. dlg reliably surrounded terminal boutons in all genotypes examined, suggesting that *Hsc70-5* knockdown did not affect SSR morphogenesis (**Fig. 1F**). Furthermore, quantification of the number, intensity, and size of glutamate receptor cluster per synapse revealed no postsynaptic phenotypes (**Fig. 1G**).

We next assessed defects in presynaptic maturation by quantifying the percentage of PSDs unopposed by Azs. To this aim, we analyzed the fraction of PSDs apposed by Az component brp that reliably localizes to mature synapses. Az apposed to newly forming PSDs usually recruit detectable brp puncta within 2-4 h of their formation and more than 95% of all glutamatergic synapses are apposed by brp [20]. A higher percentage of brp-negative synapses is indicative of neurodegenerative or neurodevelopmental defects [21]. elav>*Hsc70-5^KK100233^* larvae displayed defects in the apposition of PSDs and Azs. Twice as many unapposed, putatively immature synapses were detected (**Fig. 1G**). This defect might be caused by impairments in presynaptic maturation or stabilization.

Defects in microtubule (MT) cytoskeletal organization have been associated with PD models [22,23] in particular, and with loss of synaptic mitochondria in general [24,25]. The *Drosophila* homolog of the *LRRK2* (leucine-rich repeat kinase 2), linked to familial and sporadic PD, controls MT stability at *Drosophila* NMJs by suppressing futsch function [23]. Additionally, a decreased number of MT loops, which is indicative of stable MT, has been observed [24]. In this study, fewer MT loops were detected in terminal boutons at the NMJs of elav>*Hsc70-5^KK100233^* larvae (**Fig. 2A and S3**). In addition to defective MT morphology, we observed a reduction in the percentage of boutons connected to the stable MT network (**Fig. S3**). This impaired long-range intracellular transport. Next, we employed the synaptic footprint assay and examined neuronal membranes by labeling hrp. Synaptic footprints are biomarkers for late-stage synapse retraction [26], while hrp inhomogeneity has been associated with early-stage synapse disassembly [25]. We visualized the presynaptic compartment by labeling for the membrane marker hrp and the SV marker VGlut. Neither synaptic footprints nor hrp inhomogeneity was detected in response to *Hsc70-5* knockdown (**Fig. 2B**) suggesting that elav>*Hsc70-5^KK100233^* larvae represented an early stage of pathology with no evidence of synapse dismantlement.

***Functional consequences of loss of Hsc70-5 function.***

Next, we investigated whether the aforementioned morphological changes affect synaptic function. To examine whether symptomatic elav>*Hsc70-5^KK100233^* larvae have impaired synaptic transmission, we recorded evoked excitatory junctional potentials (eEJPs) by using current-clamp recording at NMJ from muscle 6 in segment A5. The amplitude of eEJPs in elav>*Hsc70-5^KK100233^* was reduced compared to control (**Fig. 3A**). We also recorded spontaneous events (**Fig. 3B and 3C**) and the response of nerve terminals during and following high-frequency stimulations (**Fig. 3D-F**). We noted a decrease in the amplitude of miniature excitatory junctional potential (mEJP) and an increase in their frequency in elav>*Hsc70-5^KK100233^* larvae compared to control. The ratio of eEJP to mEJP or quantal content was reduced in mutants compared to control (**Fig. 3C**). 10 Hz stimulation at mutant terminals caused no changes in EJP amplitude during a 10 Hz stimulation paradigm (**Fig. 3D and 3E**). However, a drastic time-dependent increase in failure in mutant compared to control was observed (**Fig. 3D and 3F**).

***Overexpression of* Drosophila Hsc70-5 *and human* HSPA9 *rescued* Hsc70-5 *RNAi-induced defects.***

HSPA9 consists of two principal domains: an ATPase binding domain and a substrate-binding domain. Rare variants R126W, A476T and P509S are identified in PD patients [17,27,28]. R126W localizes to the ATPase domain and, A476T and P509S respectively, to the substrate-binding domain of HSPA9 [17,27,28]. To investigate the functional relevance of these sequence variants, we performed genetic rescue experiments in the elav>*Hsc70-5^KK100233^* background using *Drosophila* UAS*-Hsc70-5*. Pan-neuronal expression (elav-Gal4) of *Hsc70-5^KK100233^* alone or in combination with a second UAS construct expressing *lacZ* at 25°C, to control for Gal4 dilution, caused lethality at the pupal stage (**Fig. 4A**). Co-overexpression of *Hsc70-5* with *Hsc70-5^KK100233^* restored adult viability (**Fig. 4A**). Since human *HSPA9* and *Drosophila Hsc70-5* have high sequence similarity [14], we examined if expression of *HSPA9* can rescue pupal lethality. Overexpression of *WT* *HSPA9* rescued pupal lethality, suggesting functional homology between the *Drosophila Hsc70-5* and human *HSPA9* (**Fig. 4A**). All investigated sequence variants, R126W, A476T and P509S (*HSPA9^A476T^*, *HSPA9^P509S^* and *HSPA9^R126W^* respectively) [17,27,28] failed to rescue pupal lethality (**Fig. 4A**). Overexpression of either *Hsc70-5* or *WT HSPA9* concomitantly with *Hsc70-5^KK100233^* successfully rescued righting reflex and lowered righting time to control levels (**Fig. 4B**). In contrast, *HSPA9^R126W^*, *HSPA9^A476T,^* and *HSPA9^P509S^* were unable to revert the righting defect (**Fig. 4B**). Next, we co-overexpressed *Hsc70-5* or *HSPA9* with *Hsc70-5^KK100233^* and performed mitochondrial morphological analysis at the larval NMJ. Overexpression of *Hsc70-5* or *WT HSPA9* rescued number, area fraction of mitochondria at the NMJ. Moreover, normal mitochondrial size and morphology were restored. In contrast, *HSPA9^R126W^, HSPA9^A476T^*^,^ and *HSPA9^P509S^* failed to rescue these phenotypes (**Fig. 4C and 4D**). These results suggested that *Hsc70-5* or *WT HSPA9* rescued pupal lethality and larval locomotion defects by restoring Hsc70-5 function.

We utilized the Gal4/Gal80 system (elav>*Hsc70-5^KK100233^*,*tub-GAL80^ts^*) to achieve late-onset conditional knockdown. Raising larvae at 18°C before transferring them to 25°C for Gal4 expression 5 d AEL (after egg laying) prevents pupal lethality. We thus could analyze behavioral defects in flies. 4-d post-eclosion, elav>*Hsc70-5^KK100233^*,*tub-GAL80^ts^* flies exhibited severe climbing defects and abnormal wing posture (**Fig. 4E and 4F**). We referred to these flies as symptomatic (**Table S1**). Late-onset knockdown of *Hsc70-5* caused a reduction of ATP levels in fly heads (**Fig. 4G**). Overexpression of either *Hsc70-5* or *WT HSPA9* in the elav>*Hsc70-5^KK100233^*,*tub-GAL80^ts^* background improved climbing ability, wing posture, and restored ATP levels in symptomatic model (**Fig. 4E-G**). Overexpression of *HSPA9^R126W^*, *HSPA9^A476T^* and *HSPA9^P509S^* variants did not rescue any of the aforementioned defects (**Fig. 4E-G**). ATP availability is crucial for the regulation of intracellular calcium at the presynapse, in particular, upon exposure to temperature-dependent cellular stress [29-31]. Furthermore, ATP is required for reversal of membrane potential following depolarization and propagation of action potentials along axons [32]. Flies with mutations in mitochondrial protein mt:CoIII (mitochondrial Cytochrome c oxidase subunit III) have been reported to suffer from temperature-induced paralysis [33]. We assayed flies at 39.5°C. elav>*Hsc70-5^KK100233^*,*tub-GAL80^ts^* flies paralyzed faster than control flies (**Fig. 4H**). This defect was rescued by overexpression of either *Hsc70-5* or *WT HSPA9* but not *HSPA9^R126W^, HSPA9^A476T^* or *HSPA9^P509S^* variants (**Fig. 4H**).

***Downregulation of autophagy was protective.***

ATP and proper mitochondrial function are important to maintain synaptic transmission at increased temperatures [29]. Mutants with compromised mitochondrial function paralyze faster compared to controls. We thus performed a candidate-based screen to identify modifiers of Hsc70-5 loss of function (**Fig. 5A**). Downregulation of synaptic proteins involved in neurotransmission and endocytic machinery enhanced heat-stress induced paralysis observed upon loss of *Hsc70-5*. Knockdown of Parkinson disease-associated *dj-1β* and *Lrrk*, as well as components of retrograde transport machinery, were suppressors of *Hsc70-5* knockdown-induced paralysis.

We identified five members of the autophagy machinery *Atg1, Atg5, Atg7, Atg12, and Atg101* as suppressors of Hsc70-5 loss of function induced paralysis (**Fig. 5A**). To verify the RNAi efficiency of modifiers isolated in the screen, we tested the *Atg1, Atg5, Atg7, Atg12, and Atg101* RNAi strains for their ability to inhibit starvation-induced autophagy (**Fig. 5B**). Overexpression of RNAi against *Atg1, Atg5, Atg7, Atg12, and Atg101* in the larval fat body using a mosaic system [34,35] for genetic analysis in cells expressing GFP completely suppressed starvation-induced autophagy (**Fig. 5B**). After validation of these constructs, we tested if knockdown of autophagy-related genes could ameliorate phenotype caused by loss of *Hsc70-5*. We used UAS-RNAi strains against *lmd* and *w* (white) with *Hsc70-5^KK100233^* (*Hsc70-5^KK100233^*+*w-RNAi* and *Hsc70-5^KK100233^*+*lmd-RNAi*) as controls to compare larvae bearing *Hsc70-5^KK100233^* in combination with a second UAS site expressing RNAi against autophagic components. Pan-neuronal expression of RNAi strains targeting components of the autophagic machinery, *Atg1, Atg5, Atg7, Atg12, and Atg101* in the elav>*Hsc70-5^KK100233^* background restored the righting reflex to control levels (**Fig. 5C**). Next, we extended the analysis to flies. As previously demonstrated, 4-d-old elav>*Hsc70-5^KK100233^*,*tub-GAL80^ts^* symptomatic flies displayed severe defects in climbing and wing posture (**Fig. 5D and 5E**) [14]. Knockdown of autophagic components reversed impairments in locomotion, wing posture and ATP levels (**Fig. 5D-F**) induced by *Hsc70-5* knockdown in young flies. Knockdown of autophagy genes alone did not reveal any significant differences compared to controls (**Fig. 5C-F**). These results suggested that inhibiting autophagy was sufficient to rescue *Hsc70-5* knockdown associated impairments in larval and adult symptomatic models.

***Autophagy induction in symptomatic flies exacerbated* Hsc70-5 *knockdown induced defects.***

Loss of Hsc70-5 function has been associated with increased mitophagy in *Drosophila* and human fibroblasts [14]. Consistent with a protective role for autophagy, *PRKN* overexpression is sufficient to rescue alterations in mitochondrial morphology and increased apoptosis [36] caused by *HSPA9* silencing in HeLa cells and the dopaminergic SH-SY5Y cell line. To address whether promoting autophagy was detrimental or protective *in vivo*, we at first modulated autophagic flux genetically. Quantification of larval locomotion revealed that pan-neuronal overexpression of *Atg1* at 25°C was sufficient to cause a sluggish righting reflex. Concomitant overexpression of elav>*Hsc70-5^KK100233^* did not further exacerbate this phenotype (**Fig. 6A**). Using the Gal4/Gal80 system, we investigated the effect of *Atg1* overexpression on *Hsc70-5* knockdown related phenotypes in flies by performing the longevity assay (**Fig. 6B**). Knockdown of *Hsc70-5* caused a significant reduction in median and maximum life expectancy compared to control flies. *Atg1* overexpression in the control background enhanced median but not maximum survival. Notably, concomitant overexpression of *Atg1* with *Hsc70-5^KK100233^* caused a reduction in both median and maximum lifespan compared to *Hsc70-5^KK100233^* alone (**Fig. 6B**). *Atg1* overexpression in the elav>*Hsc70-5^KK100233^*,*tub-GAL80^ts^* background exacerbated the loss of *Hsc70-5*-induced climbing impairment (**Fig. 6C**); however, a slight improvement was observed in wing phenotypes (**Fig. 6D**). *Atg1* overexpression alone did not impair adult locomotion of 4-d-old flies examined using the climbing ability nor induce abnormal wing phenotypes (**Fig. 6C and 6D**). These results suggested that overexpression of *Atg1* did not modify *Hsc70-5* knockdown induced defects at the larval stage but reduced lifespan and climbing ability in flies.

Rapamycin has been used to induce autophagy in *Drosophila* [37]. Feeding rapamycin was sufficient to enhance autophagy as demonstrated by increased accumulation of autophagy markers Atg8a and relative levels of Atg8a-II, in addition to reduced levels of ref(2)P, homolog of mammalian SQSTM1/p62 (**Fig. 6E and 6F**). *Hsc70-5* knockdown caused the same effects in fly heads (**Fig. 6E and 6F**). We investigated whether supplementing rapamycin could further enhance autophagy caused by *Hsc70-5* knockdown. Quantification of western blots showed a further increase in the accumulation of total Atg8a and relative levels of Atg8a-II with concomitant reduction of ref(2)P (**Fig. 6E and 6F**). Rapamycin treatment prolonged lifespan in control flies but caused a reduction in elav>*Hsc70-5^KK100233^*,*tub-GAL80^ts^* flies (**Fig. 6G**). Moreover, rapamycin treatment exacerbated the climbing impairment and wing phenotypes in symptomatic flies (**Fig. 6H and 6I**).

*PINK1* has been implicated in familial PD. It regulates the degradation of old and dysfunctional mitochondria in health and upon exposure to the mitochondrial uncoupler CCCP [6-8,38,39]. Hence, we examined if *Pink1* overexpression could modulate the loss of *Hsc70-5* phenotypes. *Pink1* overexpression was sufficient to induce a sluggish righting reflect at the larval stage (**Fig. 6J**). In elav>*Hsc70-5^KK100233^* larvae it rescued locomotion defects (**Fig. 6J**). *Pink1* overexpression in the elav>*Hsc70-5^KK100233^*,*tub-GAL80^ts^* genetic background reduced lifespan (**Fig. 6K**) and exacerbated the climbing impairment and wing phenotypes in symptomatic adult flies (**Fig. 6L and 6M**).

**Atg1 *knockdown rescued synaptic mitochondrial loss and synaptic defects observed in symptomatic larvae.***

*Atg1* is required for the initiation of autophagosome formation [40]. We thus characterized the cellular changes caused by modulation of *Atg1* expression in the elav>*Hsc70-5^KK100233^* background. *Atg1* knockdown reversed the loss of mitochondria observed at the NMJs of elav>*Hsc70-5^KK100233^* larvae (**Fig. 7A**) and rescued the decrease in mitochondria area fraction, mitochondrial number, size, and altered morphology at the NMJ in elav>*Hsc70-5^KK100233^* larvae (**Fig. 7B**).

NMJs of early symptomatic elav>*Hsc70-5^KK100233^* larvae were characterized by reduced presence of SV marker proteins and Az components, impaired synapse maturation, and alterations in the MT cytoskeleton. We next assessed MT abundance as a marker for synapse stability. *Atg1* knockdown restored usual MT abundance in elav>*Hsc70-5^KK100233^* larvae (**Fig. 7C**). Furthermore, *Atg1* knockdown in elav>*Hsc70-5^KK100233^* larvae alleviated impairments in VGlut (**Fig. 8A and 8B**) and brp abundance (**Fig. 8C and 8D**), and synapse maturation impairment (**Fig. 8E and 8F**). Overexpression of *Atg1* failed to rescue defects in MT abundance (**Fig. 7C**), SV proteins abundance (**Fig. 8A and 8B**), and synaptic maturation defects caused by loss of *Hsc70-5* at the NMJ (**Fig. 8E and 8F**). These findings indicated that restoring mitochondrial mass by *Atg1* knockdown could rescue loss of *Hsc70-5* induced synaptic defects*.*

***Autophagy suppression was protective against oxidative stress.***

Next, we tested whether knockdown of *Hsc70-5* enhances vulnerability to oxidative stress. We treated young flies with hydrogen peroxide (H_2_O_2_). H_2_O_2_ induces generalized oxidative stress which is less specific than the effects of the mitochondrial complex inhibitors like rotenone or paraquat [41]. *dj-1β* and *Lrrk* are crucial for protection against oxidative stress [41,42]. H_2_O_2_ has also been shown to reduce the lifespan of flies that display a reduced function of dj-1β and Lrrk. elav>*Hsc70-5^KK100233^*,*tub-GAL80^ts^* flies were more vulnerable to H_2_O_2_ treatment than control flies (**Fig. 9A**). While *Atg1* knockdown caused a minor reduction of lifespan upon exposure to oxidative stress compared to control, it proved to be effective in restoring the diminished stress resistance in the elav>*Hsc70-5^KK100233^*,*tub-GAL80^ts^* background (**Fig. 9A**). We thus concluded that suppression of autophagy in the elav>*Hsc70-5^KK100233^,tub-GAL80^ts^* background might be beneficial for survival under oxidative stress.

***Autophagy suppression improved healthspan but did not extend lifespan.***

To investigate the long-term impact of *Atg1* knockdown, we examined the lifespan of flies under normal conditions (**Fig. 9B**). *Atg1* knockdown led to a reduction of the lifespan in elav>*Hsc70-5^KK100233^,tub-GAL80^ts^* flies. As a minor decrease in lifespan had been observed, we thought to assess the effect of *Atg1* knockdown at various pathological stages. *Atg1* knockdown was beneficial in 4-d-old symptomatic flies and improved locomotion. In 10-d-old flies, concomitant knockdown resulted in impairment in climbing using a less challenging locomotion assay (**Fig. 9C**). We referred to these 10-d-old flies as late-symptomatic flies (**Table S1**). We concluded that reduced autophagy flux was beneficial for improving locomotion ability in *Hsc70-5* reduced background in young flies, but impaired climbing ability and lifespan in elav>*Hsc70-5^KK100233^,tub-GAL80^ts^* flies in the long run.

**Discussion**

***Investigating the impact of rare* Hsc70-5** ***mutations* in vivo.**

Three mutations have been identified in *HSPA9* in a congenital disease termed epiphyseal, vertebral, ear, nose, plus associated finding syndrome or EVEN-PLUS [28]. Biallelic mutations are identified in three individuals from two families, including the previously reported R126W variant [17]. Functional studies investigating the impact of variant *HSPA9^P509S^* reveals a lower ATP catalysis rate and reduced chaperoning activity compared to *WT* *HSPA9* [17]. Next, morphological studies in human fibroblasts from a heterozygous carrier of variant *HSPA9^A476T^* shows mitochondrial defects compared to *WT* *HSPA9* homozygous sibling [17]. Another study investigates the effects of human variant R126W, and P509S in yeast by generating analogous substitutions in the *Saccharomyces cerevisiae* ortholog of *Hsc70-5*, the *SSC1* gene [16]. The substrate-binding domain mutation *SSC1 ^A453T^* is shown to cause mitochondrial dysfunction, enhanced ROS levels, and reduces ability to prevent aggregate formation of unfolded substrates [16].

In this study, we used a *Drosophila* model of loss of Hsc70-5 function to test the functional relevance of rare *HSPA9* mutations *HSPA9^R126W^, HSPA9^A476T^*^,^ and *HSPA9^P509S^* *in vivo*. Overexpression of human *HSPA9* and *Drosophila Hsc70-5*, in a genetic background with reduced *Hsc70-5,* unlike mutant variants *HSPA9^R126W^, HSPA9^A476T^* and *HSPA9^P509S^*, were able to rescue the loss of synaptic mitochondria, locomotion defects, abnormal wing phenotype, and reduced ATP levels. This study provided evidence that the investigated amino acid replacements in the ATPase and substrate-binding domain impaired HSPA9 function. *In vitro* overexpression studies have suggested no difference in overexpression levels or localization of the mutant variants [17] and future studies should address this in *in vivo* setting.

**Hsc70-5 *knockdown induced synaptic defects.***

Neuronal mitochondria have been implicated in diverse functions including spine formation [43], synaptic plasticity [44], and axonal branching [45]. Loss of *Miro* dramatically excludes mitochondria from distal compartments and causes a gradual time-dependent reduction in EJP amplitude following 10 Hz stimulation, although no changes are observed in EJP or mEJP amplitude under baseline conditions [24]. Similar findings are reported in *Drp1* mutants where dramatic synaptic mitochondria reduction is observed [46].

Loss of neuronal mitochondria as a consequence of *Hsc70-5* silencing caused a reduction in EJP and mEJP amplitudes and quantal content (**Fig. 3A-C**). There was an increase in the frequency of mEJP events (**Fig. 3C**), possibly due to the elevation of intracellular calcium levels following loss of *Hsc70-5*. More frequent synaptic failure was observed upon stimulation at 10 Hz (**Fig. 3F**). Failed responses that are caused by the depletion of synaptic vesicles are preceded by a gradual decline in EJP amplitude [46]. However, in our case, the amplitude of the remaining EJPs was not affected. Thus, failures may instead be caused by impaired propagation of action potentials as described for *Atpα* mutant [32].

Cellular analysis of larval NMJ in *Hsc70-5* knockdown animals did not reveal gross impairments in the morphology of distal compartments (**Fig. S2**) or inhomogeneity of presynaptic membrane or synaptic footprints (**Fig. 3B**) that are biomarkers for early and late stages of synapse disassembly, respectively [25,26]. Importantly, defects in the availability of synaptic components, such as Csp, VGlut, and brp (**Fig. 1**), were observed at a stage in which no major neurodegeneration is noted [47]. Hence, the protective effects of blocking mitophagy described in this study were likely related to mitochondrial function rather than susceptibility to cell death. At this stage of disease progression, RNAi against *Atg1* was sufficient to reverse defects in development, regulation of synaptic vesicles, synaptic terminal stability, and function (**Fig. 7 and 8**). Furthermore, *Atg1*-RNAi restored mitochondria abundance. In addition, it also reversed the only putatively degenerative change we found in *Hsc70-5* knockdown larvae: MT cytoskeletal alterations (**Fig. 7B**).

***Epistatic interaction of* Hsc70-5 *with the autophagic machinery.***

Mitophagy, a type of macroautophagy that targets specifically mitochondria, is increased upon partial loss of Hsc70-5 function in *Drosophila* and HSPA9 function in human fibroblasts [14]. Increased autophagic flux can be likened to a double-edged sword that protects neurons from chronic oxidative stress but accelerate mitochondrial loss under conditions of premature mitophagy or impaired mitochondrial biogenesis [13,48]. The beneficial effects of *Park* overexpression in mouse embryonic fibroblasts treated with siRNA against *Hspa9* are dependent on intact autophagic machinery, suggesting a potential therapeutic benefit of upregulating autophagy [17]. However, the interpretation of suppressed apoptosis in HEK293 cells and tumor-derived SH-SY5Y cells is complicated by the fact that (like most tumor-derived culture cells) these cells primarily generate ATP via aerobic glycolysis and are therefore not dependent on oxidative phosphorylation [49]. Thus, the balance between costs and benefits of effective mitochondria removal may differ *in vivo*.

We demonstrate that *Hsc70-5* knockdown in *Drosophila* causes a severe loss of synaptic mitochondria and cellular ATP depletion [13]. The *Drosophila* NMJ has proved to be a particularly useful model as it is easily accessible and morphologically complex. These characteristics allow us to model a highly active synaptic population that is vulnerable to impairment in intracellular trafficking because it is located very distantly to the neuronal soma. The *in vivo* data obtained in this study did not provide evidence for the therapeutic benefit of promoting autophagic flux following impaired mitochondrial function. *Atg1* overexpression, which is sufficient to induce autophagy in *Drosophila* [50] did not rescue impaired locomotion or mitochondrial mass following *Hsc70-5* knockdown in the symptomatic larval model (**Fig. 7A**). Besides, *Atg1* overexpression did not reverse alterations in synaptic development at the larval NMJ (**Fig. 8**). In symptomatic flies, *Atg1* overexpression exacerbated climbing defects and shortened lifespan caused by loss of *Hsc70-5*. The inability of *Atg1* to exacerbate *Hsc70-5* knockdown-associated mitochondrial abundance or cellular defects at the larval stage might be explained by the fact that overexpression of *Atg1* alone did not reduce mitochondrial abundance (**Fig. 7A**). It was also possible that the loss of mitochondria upon *Hsc70-5* knockdown was so extreme that further *Atg1* overexpression was unable to exacerbate the already severe defect (**Fig. 7 and 8**).

The analysis of *Atg1* overexpression in larval stages was complicated by the fact that *Atg1* overexpression alone caused a sluggish righting phenotype (**Fig. 6A**). *Pink1* overexpression alone also caused a sluggish righting phenotype in larvae and reduced life expectancy in adult flies (**Fig. 6**). Nevertheless, both *Atg1* and *Pink1* overexpression exacerbated loss of *Hsc70-5-*associated defects in locomotion and longevity in adult flies. Future studies need to address the interaction between *Hsc70-5* and regulators of mitochondrial quality control such as *Pink1* and *park* and proteins involved in mitochondrial dynamics in more detail.

Using a functional genetic screen, we identified that knockdown of autophagy-related genes in elav>*Hsc70-5^KK100233^*,*tub-GAL80^ts^* flies rescued climbing defects, abnormal wing posture, and ATP levels in 4-d-old flies (**Fig. 5B**). However, knockdown of autophagy was detrimental in late-symptomatic 10-d-old flies.

How can these data be reconciled with reports suggesting that increased autophagy may be of therapeutic potential in PD [51]? Mitochondrial quality control mechanisms are very divergent. In particular, the molecular mechanisms underlying mitochondrial stress responses in *Drosophila* and mammals differ. While neither *prkn* nor *pink1* knockout mice display noticeable behavioral or morphological changes under baseline conditions, loss of *park* or *Pink1* in *Drosophila* is detrimental and results in decreased lifespan and apoptotic flight-muscle degeneration [6,7,52,53]. The mammalian system may be equipped with more elaborate compensatory mechanisms and display more functional redundancy, whereas flies are particularly vulnerable to overactivation of mitophagy as evident by the extreme depletion of mitochondria from synapses (**Fig. 1B**). Similar pathological induction of autophagy has been reported for hypoxic-ischemic brain injury and two toxin-based PD models in which blockade of autophagy proved to be protective [54].

***Stimulation of autophagy under conditions of oxidative stress.***

Oxidative and nitrosative stress is associated with various neurodegenerative diseases, including PD [55,56]. Pathogenic mechanisms include excitotoxicity, endoplasmic reticulum stress, protein aggregation, and damage to mitochondria, which are generators and targets of reactive oxygen species (ROS). Flies affected by *Hsc70-5* loss displayed increased vulnerability to oxidative stress (**Fig. 9A**). These effects may be mediated through the HSPA9 interaction partner PARK7 [57]. PARK7 associated with autosomal-recessive early-onset familial PD is important for response against oxidative stress [58,59]. Loss of PARK7 renders cells vulnerable to oxidative stress [57,60,61]. Notably, the interaction between HSPA9 and PARK7 is vital for the control of oxidative stress in hematopoietic stem cells [57].

Cross-talk between autophagy and ROS/nitrosative stress in the context of cell signaling and pathological protein damage is considered a significant obstacle for the therapeutic modulation of autophagy [62,63]. Thus, it is noteworthy that suppression of autophagy was beneficial in the context of combined mitochondrial dysfunction and oxidative stress in the symptomatic adult model (**Fig. 7 and 9**) presented in this study. The *in vivo* evidence presented here suggested that reduced rates of autophagy might be protective for neurons compromised by pathologically increased levels of mitophagy. However, we observed that detrimental side effects exceeded protective benefits in the long-term (**Fig. 9**).

**Materials and Methods**

***Fly strains.***

Transgenic fly stocks were obtained either from Indiana University Stock Center (Bloomington, IN, USA) or Vienna *Drosophila* RNAi center unless otherwise noted. Transgenic stocks UAS-*WT HSPA9*, UAS-*HSPA9^R126W^*, UAS-*HSPA9^A476T^*, UAS-*HSPA9^P509S,^* and UAS-*Hsc70-5* were created by BestGene using integrase-mediated site-specific transgenesis at cytological position 68A4 (Fly strain BDSC 8622). cDNA constructs for *WT* *HSPA9*, *HSPA9^R126W^*, *HSPA9^A476T^*, *and HSPA9^P509S^* were received from Rejko Krüger (University of Luxembourg). The constructs were recloned and inserted into a modified pUAST attB vector (Drosophila Genomics Resource Center, 1419) (using BamHI and XhoI). The full-length *Drosophila Hsc70-5* (GM13788) was inserted into a modified pUAST attB vector. Details of fly strains have been provided in the supplementary information (Materials and Methods, **Table S2**).

***Fly culture conditions.***

Flies were raised on standard cornmeal/agar medium. To circumvent pupal lethality caused by *Hsc70-5* knockdown and analyze behavioral defects in adult flies, we utilized the Gal4/Gal80 system (elav>*Hsc70-5^KK100233^,tub-GAL80^ts^*). This allowed us to achieve late-onset conditional knockdown by raising larvae at 18°C before transferring them to 25°C at 5 d AEL. Flies were kept at 25°C during development for analysis of wing phenotype, climbing defects, longevity, ATP levels from heads, and temperature-induced paralysis at appropriate ages.

***Electrophysiology.***

Electrophysiological recordings were performed essentially as previously described [19,25]**.**

***Staining and imaging larval neuromuscular junctions.***

Dissection and labeling of size-matched mid-3^rd^ instar larvae were performed using previously described protocols [25]. Antibodies were obtained from Developmental Studies Hybridoma Bank unless otherwise noted. Larvae expressing GFP were fixed for 3 min (4% paraformaldehyde in phosphate-buffered saline (PBS; Thermo Fisher Scientific, 10010023). Goat anti-hrp-Cy3 antibody was obtained from Dianova GmBH (323-165-021). Primary antibodies were used in the following dilutions: mouse anti-brp (1:100; nc82), mouse anti-Csp (1:150; 6D6), rabbit anti-VGlut (1:1000; a gift from Hermann Aberle, Max Planck Institute for Developmental Biology), mouse anti-dlg (1:100; 4F3), and mouse anti-futsch (1:100; 22C10), mouse anti-GluR (1:2000; a gift from Stephan Sigrist, Free University of Berlin). Confocal imaging of NMJ 4 at Segment A5 of mid-3^rd^ instar larvae was performed using a Zeiss LSM 710 microscope (Carl Zeiss AG, Oberkochen, Germany) using a 40× Plan-Apochromat 1.4 N.A. oil objective. A voxel dimension of (x/y/z) 100×100×500 nm was utilized. The pinhole size was 1 Airy Disc. Images were scaled by a factor of 2 before Gaussian blur filtering was applied (pixel radius=2). Gamma values were set to 0.75. For quantitative comparisons of intensities, standard imaging settings were chosen that avoided oversaturation. ImageJ Software Version 1.43e (National Institutes of Health, Bethesda, MD, USA) was used for image processing.

***Morphological analysis.***

Analyses of mitochondria and NMJs were performed as previously described [14]. The area fraction occupied by mitochondria was used as the index of mitochondrial mass. To quantify futsch loops, the number of loops located to the two most distal boutons at each terminal of the NMJ was scored. The total number of loops in these regions was normalized to the number of terminals.

***Fat body assay for investigating autophagy.***

Assay to validate functionality of UAS-*Atg1* overexpression or RNAi constructs against autophagy-related proteins were performed using a mosaic genetic analysis in larval fat body cells essentially as previously described [34,35]. Starvation of 2^nd^ instar stage larvae was performed for 5-6 h in fresh empty vials on a filter paper soaked with H_2_O. For imaging, 2^nd^ instar larvae that were well fed or starved for 4-5 h were cut open and turned inside out like a ‘sock’ before removal of fat bodies. Fat body tissues were stained with DAPI for 2 min, briefly washed in PBS, and mounted on a glass slide using Vectashield and imaged immediately using a Leica SP5 II confocal imaging system (Leica Camera AG, Wetzlar, Germany) using a 40× Plan-Apochromat 1.4 N.A. oil objective. The entire procedure from fat body isolation to imaging was completed within 30 min.

***ATP measurements.***

ATP levels in head homogenates were measured and normalized using a luciferase-based bioluminescence assay as described earlier [13]. Five heads of female flies were homogenized in 6 M guanidine-HCl (Sigma-Aldrich, SRE0066) and frozen in liquid nitrogen. Next, samples were boiled for 3 min, cleared by centrifugation at 14000 g for 5 min, and diluted to measure protein concentration (1:10 diluted samples, Bradford Assay Kit; Sigma-Aldrich, B6916) and ATP level (1:2000 diluted samples, ATP Determination Kit Sensitive Assay; Biaffin GmbH & Co KG, LBR-P010). ATP levels were normalized to the protein concentration.

***Locomotion analysis.***

Climbing assays were, unless otherwise noted, conducted as previously described [14]. Climbing male flies was monitored by analyzing their performance to climb 6 cm (challenging assay, used in **Fig. 4E and 5D**) or 3 cm (less challenging assay used in **Fig. 9C** for 10-d-old flies) within 14 s. A successful attempt was scored as 1, and failure to reach the top as 0. Each fly was assessed three times to calculate the average climbing score. At least 40 flies per genotype were analyzed. Larval locomotion was investigated by examining larvae crawling speed and righting assay. Larvae locomotion speed was quantified as previously described [25].

***Longevity assay.***

*Drosophila* larvae were transferred from 18°C to 25°C to boost UAS-transgene expression 5 d AEL. Adult male flies were maintained at 25°C in groups of 20 or fewer. 100 flies were assayed per genotype. No anesthesia was used in survival experiments.

***Rapamycin treatment.***

Rapamycin (LC Laboratories, 53123-88-9) was dissolved in ethanol and added to standard fly food (Meidi LLC, V100) at appropriate concentrations (0.1 and 200 μM). For control food (0 μM), ethanol alone was added. 5 d AEL, larvae were transferred from 18°C to 25°C to induce transgene expression on 0 μM and 0.1 μM rapamycin food. Newly hatched male flies were continually maintained in 0 μM and 200 μM rapamycin food at 25°C for longevity assay.

***Western blot.***

Western blot analysis was performed on 20 µg samples of 4-d-old fly head protein using antibodies for rabbit anti-Atg8a and rabbit anti-ref(2)P (1:1000; a gift from Gábor Juhász, Eotvos Lorand University) and mouse anti-TUBB/β-tubulin (1:1000; Developmental Studies Hybridoma Bank, E7) using chemiluminescent detection.

***Statistical analysis.***

Statistical tests were performed using PAST.exe software (http:// folk.uio.no/ohammer/past/index.html) unless otherwise noted. The standard error of mean (SEM) and standard deviation (SD) are shown as a box and black line. Data were first tested for normality by using the Shapiro-Wilk test (a=0.05). Normally distributed data were analyzed either by Student’s t-test (two groups) and Bonferroni comparison to adjust the P value or by a one-way analysis of variance followed by a Tukey-Kramer post-test for comparing multiple groups. Nonnormal distributed data were analyzed by either a Mann–Whitney test (two groups) or Bonferroni comparison to adjust P value or a Kruskal-Wallis H-test followed by a Dunn’s test for comparisons between multiple groups. Statistical significance was defined as P<0.05.

**Acknowledgement**

We thank the Developmental Studies Hybridoma Bank maintained by the University of Iowa for antibodies. We are grateful to the Bloomington and VDRC Stock Centers as well as all researchers not mentioned explicitly who made their stocks available via the Bloomington Stock Center. We thank Raphael Zinser for excellent assistance. We thank the DKFZ Light Microscopy Facility. We thank the Fritz Thyssen Foundation (grant 10.12.1.192 awarded to TMR), the Hertie Foundation (for stipends awarded to JZ, SBH, NV), the Chica and Heinz Schaller Foundation (for supporting SBH, NMD, TRJ), and Max Planck Society (for supporting TMR).

**Disclosure statement**

All authors declare no conflict of interest.

**References**

1. Dauer W, Przedborski S. Parkinson's disease: mechanisms and models. Neuron. 2003 Sep 11;39(6):889-909.

2. Picconi B, Piccoli G, Calabresi P. Synaptic dysfunction in Parkinson's disease. Advances in experimental medicine and biology. 2012;970:553-72.

3. Bras J, Guerreiro R, Hardy J. SnapShot: Genetics of Parkinson's disease. Cell. 2015 Jan 29;160(3):570-570 e1.

4. Miura E, Hasegawa T, Konno M, et al. VPS35 dysfunction impairs lysosomal degradation of alpha-synuclein and exacerbates neurotoxicity in a Drosophila model of Parkinson's disease. Neurobiol Dis. 2014 Nov;71:1-13.

5. Misgeld T, Schwarz TL. Mitostasis in Neurons: Maintaining Mitochondria in an Extended Cellular Architecture. Neuron. 2017 Nov 1;96(3):651-666.

6. Clark IE, Dodson MW, Jiang C, et al. Drosophila pink1 is required for mitochondrial function and interacts genetically with parkin. Nature. 2006 Jun 29;441(7097):1162-6.

7. Park J, Lee SB, Lee S, et al. Mitochondrial dysfunction in Drosophila PINK1 mutants is complemented by parkin. Nature. 2006 Jun 29;441(7097):1157-61.

8. Geisler S, Holmstrom KM, Treis A, et al. The PINK1/Parkin-mediated mitophagy is compromised by PD-associated mutations. Autophagy. 2010 Oct;6(7):871-8.

9. Thomas KJ, McCoy MK, Blackinton J, et al. DJ-1 acts in parallel to the PINK1/parkin pathway to control mitochondrial function and autophagy. Hum Mol Genet. 2011 Jan 01;20(1):40-50.

10. Burchell VS, Nelson DE, Sanchez-Martinez A, et al. The Parkinson's disease-linked proteins Fbxo7 and Parkin interact to mediate mitophagy. Nat Neurosci. 2013 Sep;16(9):1257-65.

11. Devi L, Raghavendran V, Prabhu BM, et al. Mitochondrial import and accumulation of alpha-synuclein impair complex I in human dopaminergic neuronal cultures and Parkinson disease brain. J Biol Chem. 2008 Apr 04;283(14):9089-100.

12. DuBoff B, Feany M, Gotz J. Why size matters - balancing mitochondrial dynamics in Alzheimer's disease. Trends Neurosci. 2013 Jun;36(6):325-35.

13. Zhu J, Wang KZ, Chu CT. After the banquet: mitochondrial biogenesis, mitophagy, and cell survival. Autophagy. 2013 Nov 1;9(11):1663-76.

14. Zhu JY, Vereshchagina N, Sreekumar V, et al. Knockdown of Hsc70-5/mortalin induces loss of synaptic mitochondria in a Drosophila Parkinson's disease model. PLoS One. 2013;8(12):e83714.

15. Wadhwa R, Ryu J, Ahn HM, et al. Functional significance of point mutations in stress chaperone mortalin and their relevance to Parkinson disease. J Biol Chem. 2015 Mar 27;290(13):8447-56.

16. Goswami AV, Samaddar M, Sinha D, et al. Enhanced J-protein interaction and compromised protein stability of mtHsp70 variants lead to mitochondrial dysfunction in Parkinson's disease. Hum Mol Genet. 2012 Aug 1;21(15):3317-32.

17. Burbulla LF, Schelling C, Kato H, et al. Dissecting the role of the mitochondrial chaperone mortalin in Parkinson's disease: functional impact of disease-related variants on mitochondrial homeostasis. Hum Mol Genet. 2010 Nov 15;19(22):4437-52.

18. Knight D, Xie W, Boulianne GL. Neurexins and neuroligins: recent insights from invertebrates. Molecular neurobiology. 2011 Dec;44(3):426-40.

19. Zhang YV, Hannan SB, Kern JV, et al. The KIF1A homolog Unc-104 is important for spontaneous release, postsynaptic density maturation and perisynaptic scaffold organization. Scientific reports. 2017 Mar 27;7:38172.

20. Rasse TM, Fouquet W, Schmid A, et al. Glutamate receptor dynamics organizing synapse formation in vivo. Nat Neurosci. 2005 Jul;8(7):898-905.

21. Kern JV, Zhang YV, Kramer S, et al. The kinesin-3, unc-104 regulates dendrite morphogenesis and synaptic development in Drosophila. Genetics. 2013 Sep;195(1):59-72.

22. Lee S, Imai Y, Gehrke S, et al. The synaptic function of LRRK2. Biochemical Society transactions. 2012 Oct;40(5):1047-51.

23. Lee S, Liu HP, Lin WY, et al. LRRK2 kinase regulates synaptic morphology through distinct substrates at the presynaptic and postsynaptic compartments of the Drosophila neuromuscular junction [Research Support, N.I.H., Extramural Research Support, Non-U.S. Gov't]. J Neurosci. 2010 Dec 15;30(50):16959-69.

24. Guo X, Macleod GT, Wellington A, et al. The GTPase dMiro is required for axonal transport of mitochondria to Drosophila synapses. Neuron. 2005 Aug 4;47(3):379-93.

25. Fuger P, Sreekumar V, Schule R, et al. Spastic paraplegia mutation N256S in the neuronal microtubule motor KIF5A disrupts axonal transport in a Drosophila HSP model. Plos Genet. 2012;8(11):e1003066.

26. Eaton BA, Fetter RD, Davis GW. Dynactin is necessary for synapse stabilization. Neuron. 2002 May 30;34(5):729-41.

27. De Mena L, Coto E, Sanchez-Ferrero E, et al. Mutational screening of the mortalin gene (HSPA9) in Parkinson's disease. Journal of neural transmission (Vienna, Austria : 1996). 2009 Oct;116(10):1289-93.

28. Royer-Bertrand B, Castillo-Taucher S, Moreno-Salinas R, et al. Mutations in the heat-shock protein A9 (HSPA9) gene cause the EVEN-PLUS syndrome of congenital malformations and skeletal dysplasia. Scientific reports. 2015;5:17154.

29. Klose MK, Boulianne GL, Robertson RM, et al. Role of ATP-dependent calcium regulation in modulation of Drosophila synaptic thermotolerance. J Neurophysiol. 2009 Aug;102(2):901-13.

30. Kawasaki F, Felling R, Ordway RW. A temperature-sensitive paralytic mutant defines a primary synaptic calcium channel in Drosophila. J Neurosci. 2000 Jul 1;20(13):4885-9.

31. Rikhy R, Kamat S, Ramagiri S, et al. Mutations in dynamin-related protein result in gross changes in mitochondrial morphology and affect synaptic vesicle recycling at the Drosophila neuromuscular junction. Genes Brain Behav. 2007 Feb;6(1):42-53.

32. Trotta N, Rodesch CK, Fergestad T, et al. Cellular bases of activity-dependent paralysis in Drosophila stress-sensitive mutants. J Neurobiol. 2004 Sep 05;60(3):328-47.

33. Liu W, Gnanasambandam R, Benjamin J, et al. Mutations in cytochrome c oxidase subunit VIa cause neurodegeneration and motor dysfunction in Drosophila. Genetics. 2007 Jun;176(2):937-46.

34. Erdi B, Nagy P, Zvara A, et al. Loss of the starvation-induced gene Rack1 leads to glycogen deficiency and impaired autophagic responses in Drosophila. Autophagy. 2012 Jul 1;8(7):1124-35.

35. Nagy P, Varga A, Kovacs AL, et al. How and why to study autophagy in Drosophila: it's more than just a garbage chute. Methods (San Diego, Calif). 2015 Mar;75:151-61.

36. Burbulla LF, Fitzgerald JC, Stegen K, et al. Mitochondrial proteolytic stress induced by loss of mortalin function is rescued by Parkin and PINK1. Cell Death Dis. 2014;5:e1180.

37. Bjedov I, Toivonen JM, Kerr F, et al. Mechanisms of life span extension by rapamycin in the fruit fly Drosophila melanogaster. Cell metabolism. 2010 Jan;11(1):35-46.

38. Morais VA, Haddad D, Craessaerts K, et al. PINK1 loss-of-function mutations affect mitochondrial complex I activity via NdufA10 ubiquinone uncoupling. Science. 2014 Apr 11;344(6180):203-7.

39. Morais VA, Verstreken P, Roethig A, et al. Parkinson's disease mutations in PINK1 result in decreased Complex I activity and deficient synaptic function. EMBO molecular medicine. 2009 May;1(2):99-111.

40. Chang YY, Neufeld TP. Autophagy takes flight in Drosophila. FEBS letters. 2010 Apr 2;584(7):1342-9.

41. Wang D, Tang B, Zhao G, et al. Dispensable role of Drosophila ortholog of LRRK2 kinase activity in survival of dopaminergic neurons. Molecular neurodegeneration. 2008;3:3.

42. Hwang S, Song S, Hong YK, et al. Drosophila DJ-1 decreases neural sensitivity to stress by negatively regulating Daxx-like protein through dFOXO. Plos Genet. 2013 Apr;9(4):e1003412.

43. Fu ZX, Tan X, Fang H, et al. Dendritic mitoflash as a putative signal for stabilizing long-term synaptic plasticity. Nat Commun. 2017 Jun 26;8(1):31.

44. Li Z, Okamoto K, Hayashi Y, et al. The importance of dendritic mitochondria in the morphogenesis and plasticity of spines and synapses. Cell. 2004 Dec 17;119(6):873-87.

45. Courchet J, Lewis TL, Jr., Lee S, et al. Terminal axon branching is regulated by the LKB1-NUAK1 kinase pathway via presynaptic mitochondrial capture. Cell. 2013 Jun 20;153(7):1510-25.

46. Verstreken P, Ly CV, Venken KJT, et al. Synaptic mitochondria are critical for mobilization of reserve pool vesicles at Drosophila neuromuscular junctions. Neuron. 2005 Aug 4;47(3):365-378.

47. Qin G, Schwarz T, Kittel RJ, et al. Four different subunits are essential for expressing the synaptic glutamate receptor at neuromuscular junctions of Drosophila. J Neurosci. 2005 Mar 23;25(12):3209-18.

48. Dagda RK, Das Banerjee T, Janda E. How Parkinsonian toxins dysregulate the autophagy machinery. International journal of molecular sciences. 2013;14(11):22163-89.

49. Cairns RA, Harris IS, Mak TW. Regulation of cancer cell metabolism. Nature reviews Cancer. 2011 Feb;11(2):85-95.

50. Scott RC, Juhasz G, Neufeld TP. Direct induction of autophagy by Atg1 inhibits cell growth and induces apoptotic cell death. Current biology : CB. 2007 Jan 9;17(1):1-11.

51. Tan CC, Yu JT, Tan MS, et al. Autophagy in aging and neurodegenerative diseases: implications for pathogenesis and therapy. Neurobiology of aging. 2014 May;35(5):941-57.

52. Perez FA, Palmiter RD. Parkin-deficient mice are not a robust model of parkinsonism. Proc Natl Acad Sci U S A. 2005 Feb 8;102(6):2174-9.

53. Beal MF. Parkinson's disease: a model dilemma. Nature. 2010 Aug 26;466(7310):S8-10.

54. Koike M, Shibata M, Tadakoshi M, et al. Inhibition of autophagy prevents hippocampal pyramidal neuron death after hypoxic-ischemic injury. The American journal of pathology. 2008 Feb;172(2):454-69.

55. Nakamura T, Cho DH, Lipton SA. Redox regulation of protein misfolding, mitochondrial dysfunction, synaptic damage, and cell death in neurodegenerative diseases. Experimental neurology. 2012 Nov;238(1):12-21.

56. Tsang AH, Chung KK. Oxidative and nitrosative stress in Parkinson's disease. Biochimica et biophysica acta. 2009 Jul;1792(7):643-50.

57. Tai-Nagara I, Matsuoka S, Ariga H, et al. Mortalin and DJ-1 coordinately regulate hematopoietic stem cell function through the control of oxidative stress. Blood. 2014 Jan 2;123(1):41-50.

58. Kahle PJ, Waak J, Gasser T. DJ-1 and prevention of oxidative stress in Parkinson's disease and other age-related disorders. Free radical biology & medicine. 2009 Nov 15;47(10):1354-61.

59. Bonifati V, Rizzu P, Squitieri F, et al. DJ-1( PARK7), a novel gene for autosomal recessive, early onset parkinsonism. Neurological sciences : official journal of the Italian Neurological Society and of the Italian Society of Clinical Neurophysiology. 2003 Oct;24(3):159-60.

60. Meulener M, Whitworth AJ, Armstrong-Gold CE, et al. Drosophila DJ-1 mutants are selectively sensitive to environmental toxins associated with Parkinson's disease. Current biology : CB. 2005 Sep 6;15(17):1572-7.

61. Menzies FM, Yenisetti SC, Min KT. Roles of Drosophila DJ-1 in survival of dopaminergic neurons and oxidative stress. Current biology : CB. 2005 Sep 6;15(17):1578-82.

62. Lee J, Giordano S, Zhang J. Autophagy, mitochondria and oxidative stress: cross-talk and redox signalling. The Biochemical journal. 2012 Jan 15;441(2):523-40.

63. Janda E, Isidoro C, Carresi C, et al. Defective autophagy in Parkinson's disease: role of oxidative stress. Molecular neurobiology. 2012 Dec;46(3):639-61.

**Figure Legends**

**Figure 1**. Characterization of the neuromuscular junction in presymptomatic and symptomatic larval. (**A**) Average larval crawling velocity and righting reflex at L3 stage. Scale bar: 0.25 mm. (**B**) Confocal images of larval NMJ expressing mito-GFP (magenta) and labeled with hrp (green). Scale bar: 2 μm. (**C-E**) Representative synaptic boutons from NMJs of control, elav>*Hsc70-5^GD13957^* and elav>*Hsc70-5^KK100233^* larvae. Anti-hrp staining (C-E, green) was used to visualize synaptic membranes. Synaptic vesicle proteins Csp (**C**) and VGlut (**D**) and active zone component brp (**E**) were shown in magenta. Scale bar: 2 μm. (**F**) Confocal images of larval NMJ labeled with hrp (magenta) and *Drosophila* dlg (green) used to visualize the sub-synaptic reticulum (SSR). Scale bar: 5 μm, enlargement: 2 μm. (**G**) Confocal images of larval NMJ labeled with brp (magenta) and GluR (green) were used to visualize PSDs (postsynaptic densities). Arrowheads pointing out presynaptic brp labels were not detected in PSDs. Scale bar: 2 μm. The standard error of mean and standard deviation are shown as a box and a black line. * p<0.05, ** p<0.01, *** p<0.001.

**Figure 2**. Loss of Hsc70-5 function is associated with MT cytoskeleton defects. (**A**) Confocal images of larval NMJ labeled with hrp (green) and futsch (MT cytoskeleton; magenta). Scale bar: 5 μm, enlargement: 2 μm. Arrowheads pointing at NMJ loops. (**B**) Confocal images of larval NMJ labeled with hrp (gray), VGlut (gray and magenta), and dlg (green). Scale bar: 10 μm, enlargement: 2 μm.

**Figure 3**. Electrophysiological characterization of symptomatic larvae. (**A**) Representative traces of eEJPs from control and *elav>Hsc70-5^KK100233^* larvae and quantification of eEJP amplitudes. Stimulation artifacts were removed from eEJP traces. (**B**) Representative mEJP recording in control and *elav>Hsc70-5^KK100233^* larvae. Quantification of (**C**) mEJP amplitude, mEJP frequency and quantal content in control and *elav>Hsc70-5^KK100233^* larvae. (**D**) Representative traces from 10 Hz stimulation displaying five successive synaptic responses from each genotype after 1 and 10 s stimulation. Stimulation artifacts were removed for demonstration. (**E**) Quantification of eEJP amplitude in response to 10 Hz stimulation in control and *elav>Hsc70-5^KK100233^* larvae. (**F**) Quantification of % failure of eEJP in response to 10 Hz stimulation in control and *elav>Hsc70-5^KK100233^* larvae. The standard error of mean and standard deviation are shown as a box and a black line. * p<0.05, ** p<0.01.

**Figure 4**. *Drosophila Hsc70-5* and human *WT* *HSPA9* but not disease variants rescued *Hsc70-5* knockdown phenotypes. (**A**) *Hsc70-5* knockdown in combination with *lacZ* overexpression to control Gal4 dilution at 25°C caused pupal lethality. Overexpression of *Drosophila Hsc70-5* and human *WT* *HSPA9* in the *Hsc70-5^KK100233^* background unlike *HSPA9^R126W^*, *HSPA9^A476T^* and *HSPA9^P509S^* variants rescued pupal lethality. (**B**) Average larval righting reflex at L3 stage with overexpression of *Hsc70-5*, *WT* *HSPA9*, *HSPA9^R126W^*, *HSPA9^A476T^* and *HSPA9^P509S^* in the elav>*Hsc70-5^KK100233^* background. (**C**) Confocal images of larval NMJ labeled with hrp (green) and mito-GFP (magenta), and (**D**) quantification of mitochondrial parameters. Scale bar: 2 μm. (**E**) Climbing ability of 4-d-old flies, (**F**) Percentage of flies with defective wing phenotype, and (**G**) ATP levels in fly heads after expressing *Hsc70-5*, *WT HSPA9*, *HSPA9^R126W^*, *HSPA9^A476T^* and *HSPA9^P509S^* in the *elav>Hsc70-5^KK100233^* background. (**H**) *Hsc70-5* knockdown accelerated heat-shock induced paralysis in flies at 39.5°C. The co-overexpression of *Hsc70-5* and *WT* *HSPA9* unlike *HSPA9^R126W^*, *HSPA9^A476T^* and *HSPA9^P509S^* rescued this defect. The standard error of mean and standard deviation are shown as a box and a black line. * p<0.05, ** p<0.01, *** p<0.001.

**Figure 5**. A genetic screen identified autophagy-related proteins as modifiers of *Hsc70-5* knockdown phenotypes. (**A**) A genetic screen identified several modifiers that modified *Hsc70-5* knockdown-induced phenotypes. (**B**) Increase in mCherry.*Atg8a* puncta in the larval fat body following starvation. There was no difference in the expression of mCherry.*Atg8a* between GFP +ve and -ve cells in control overexpressing *lacZ* (in GFP +ve cell). Expression of RNAi against autophagy-related genes *(Atg1, Atg101 Atg5, Atg7,* and *Atg12)* inhibited starvation-induced autophagy in GFP +ve cells compared to cells in the vicinity. Scale bar: 15 μm. (**C**) Righting reflex in larvae, (**D**) Climbing ability of 4-d-old flies, (**E**) Percentage of flies with defective wing phenotype, and (**F**) ATP levels in fly heads after pan-neuronal knockdown of *Atg1, Atg101 Atg5, Atg7,* and *Atg12* in control and the elav>*Hsc70-5^KK100233^,tub-GAL80^ts^* background. The standard error of mean and standard deviation are shown as a box and a black line. * p<0.05, *** p<0.001.

**Figure 6**. Autophagy induction did not rescue symptomatic phenotypes in *Hsc70-5* knockdown flies. (**A**) Quantification of larval righting reflex, (**B**) Lifespan of flies at 25°C, (**C**) Climbing ability of 4-d-old flies and (**D**) Percentage of flies with defective wing phenotype expressing *Atg1* in the elav>*Hsc70-5^KK100233^,tub-GAL80^ts^* background. (**E**) Western blot showing levels of Atg8a, Atg8a-II, and ref(2)P in rapamycin-treated flies in the elav>*Hsc70-5^KK100233^,tub-GAL80^ts^* background. βTub56D was used as a control. (**F**) Quantifications of protein levels of Atg8a, Atg8a-II, and ref(2)P. (**G**) The lifespan of flies with rapamycin treatment in the elav>*Hsc70-5^KK100233^,tub-GAL80^ts^* background at 25°C. (**H**) The climbing ability of 4-d-old flies, and (**I**) percentage of flies with defective wing phenotype with rapamycin treatment in the elav>*Hsc70-5^KK100233^,tub-GAL80^ts^* background. (**J**) Quantification of larval righting reflex, (**K**) Lifespan of flies at 25°C, (**L**) Climbing ability of 4-d-old flies, and (**M**) percentage of flies with defective wing phenotype expressing *Pink1* in the elav>*Hsc70-5^KK100233^,tub-GAL80^ts^* background. The standard error of mean and standard deviation are shown as a box and a black line. * p<0.05, ** p<0.01, *** p<0.001.

**Figure 7**. Autophagy inhibition alleviated mitochondrial and MT defects caused by *Hsc70-5* knockdown. (**A**) Confocal images of larval NMJ expressing mito-GFP (magenta) labeled with hrp (green). Scale bar: 2 μm. (**B**) Quantification of mitochondrial area fraction, number, size, and morphology. (**C**) Confocal images of NMJ labeled with hrp (green) and futsch (magenta). Scale bar: 10 μm. The standard error of mean and standard deviation are shown as a box and a black line. * p<0.05, ** p<0.01, *** p<0.001.

**Figure 8**. Autophagy inhibition alleviated synaptic defects caused by *Hsc70-5* knockdown. (**A and C**) Confocal images of larval NMJ labeled with hrp (green), VGlut, and brp (magenta). Scale bar: 2 μm. (**B** **and** **D**) Quantification of VGlut and brp level at NMJ following *Atg1* overexpression and knockdown in elav>*Hsc70-5*^KK100233^ background. (**E**) Confocal images of larval NMJ labeled with brp (magenta) and GluR (green). Arrowheads pointing out regions where presynaptic brp labels were not detected in PSDs. Scale bar: 2 μm (**F**) Quantification of unapposed glutamate receptor fields. The standard error of mean and standard deviation are shown as a box and a black line. * p<0.05, *** p<0.001.

**Figure 9**. Concomitant *Atg*1 and *Hsc70-5* knockdown rescued longevity under oxidative stress but was detrimental upon aging in baseline conditions. (**A**) Lifespan of flies at 25°C following induction of generalized oxidative stress. Flies were fed with 5% hydrogen peroxide sucrose solution. (**B**) Lifespan of flies at 25°C on the standard diet. (**C**) The climbing ability of 4- and 10-d-old flies expressing *Atg1* knockdown in the elav>*Hsc70-5^KK100233^,tub-GAL80^ts^* background. The standard error of mean and standard deviation are shown as a box and a black line. * p<0.05, *** p<0.001.
