# Supplementary figures and images for: Autophagy inhibition rescues structural and functional defects caused by the loss of mitochondrial chaperone Hsc70-5 in Drosophila
Source: Autophagy. 2021 Jan 25;17(10):3160–74. doi: 10.1080/15548627.2020.1871211 (PMC8526020; doi:10.1080/15548627.2020.1871211)

**A**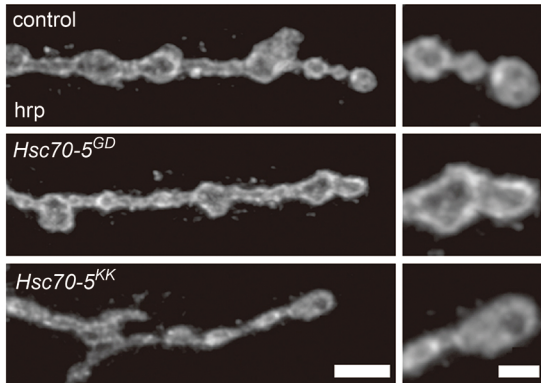**B**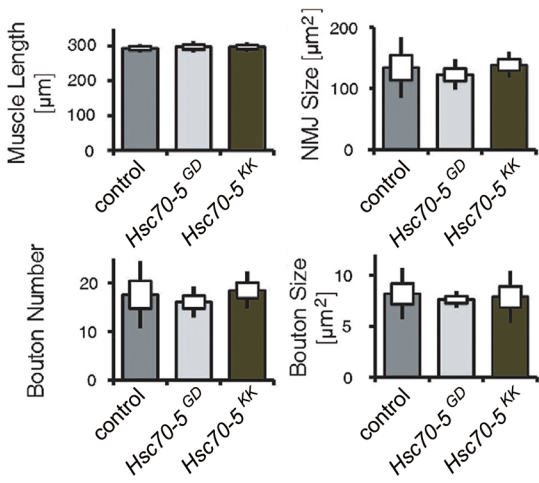

Supplement: Supplemental Material [file KAUP_A_1871211_SM4946.zip › supplement/figure 2.pdf]

**A**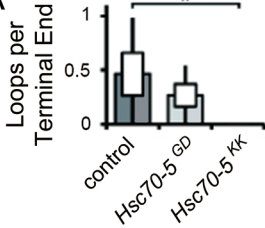**B**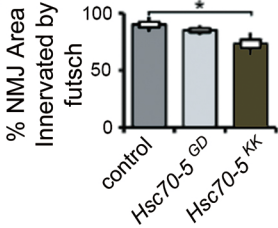

Supplement: Supplemental Material [file KAUP_A_1871211_SM4946.zip › supplement/figure s3.pdf]
